# Associations between Sleep and Mental Health in Adolescents: Results from the UK Millennium Cohort Study

**DOI:** 10.3390/ijerph19031868

**Published:** 2022-02-07

**Authors:** Jiaqi Qiu, Isabel Morales-Muñoz

**Affiliations:** 1 School of Psychology, College of Life and Environmental Sciences, University of Birmingham, Birmingham B15 2TT, UK; 2 Institute for Mental Health, School of Psychology, College of Life and Environmental Sciences, University of Birmingham, Birmingham B15 2TT, UK; I.Morales-Munoz@bham.ac.uk; 3 Mental Health Unit, Finnish Institute for Health and Welfare, FI-00271 Helsinki, Finland

**Keywords:** sleep problems, affective symptoms, emotion symptoms, conduct problems, adolescence, UK Millennium cohort

## Abstract

(1) Background: There is a growing interest in investigating the relationship between sleep and mental health development in adolescents. This study aims to further investigate this relationship by identifying the specific associations between several sleep problems in adolescents and several mental health areas, and the role of gender in these associations. (2) Methods: Data from the Millennium cohort survey containing 11,553 individuals at 13–14 years old was included. Nighttime sleep duration and bedtime during weekdays and weekends, night awakening frequency, and sleep onset latency were assessed using self-reported questionnaires. Affective symptom and emotional and behavioural problems were examined with self-reported questionnaires. (3) Results: Regression analyses and path analysis models suggested that frequent night awakening was associated with all the outcomes, and hyperactivity/inattention was the outcome that presented a higher number of significant associations with sleep patterns. Long sleep onset latency and late bedtime at school days were associated with higher risk of emotional and behavioural difficulties. Further, poor sleep seems to manifest more externally in males, while more internally in females. (4) Conclusions: Specific sleep problems should be considered when assessing mental health in adolescence, which would allow more targeted prevention and intervention strategies. Further, special attention should be given to gender differences when addressing sleep and mental health.

## 1. Introduction

Sleep is essential for our wellbeing and mental health, and it has a vital role in the development of daytime emotions and overall behaviours [1]. Therefore, over the past decades, there has been a growing interest in investigating the relationship between sleep and mental health. In particular, adolescents’ mental health has been of great interest, due to the vulnerability and sensitive nature of this transitional period [1], where individuals are at higher risk of suffering from adverse mental health problems, such as depression and/or anxiety, compared to other developmental stages [2]. For instance, young people with both self-reported and actigraphy-based sleep disturbances in adolescence are more likely to have poor mental health and impaired functioning [3]. Among all the recognisable early symptoms of mental health problems in adolescence, sleep disturbances measured by self-reported questionnaires are considered one of the key risk factors [4]. Previous research indicates of the existence of increasing sleep difficulties in adolescents measured by self-reported questionnaires [5]. In addition, severe sleep disturbances have been linked to mental and emotional dysfunction in adolescents. For instance, over 50% of adolescents aged 9–19 years old with insomnia have a comorbid psychiatric disorder, such as depression or anxiety [6]. Further, having difficulty in falling asleep measured by self-rated questionnaire is linked to attention problems, withdrawal, anxiety, and depression in adolescents [7]. Furthermore, shortened sleep duration and impaired sleep quality predicts higher anxiety, depression, and externalizing symptoms in adolescents [1]. Finally, some studies have reported that subjective late bedtime is particularly related to depression, anxiety, and suicidal or self-injury risk in monozygotic twin adolescents aged 7–12 years old [8].

However, most of the previous studies have only focused on isolated aspects of sleep and how they relate to certain areas of mental health in adolescence. However, to clearly understand the nature of the relation between sleep and mental health in adolescents, we need to further investigate concurrent sleep problems, and how they relate to a wide range of mental health problems. So far, there are few cross-sectional studies that have investigated the impact that several mental health problems exert on several mental health problems in adolescents. For instance, in a cross-sectional study based on a population of 4823 adolescents aged 11–20-years-old in China, the authors suggested that later bedtime during weekdays, maintaining sleep difficulties, and unstable sleep schedule measured by self-reported questionnaire were all related to mental health problems, including emotional symptoms, conduct problems, and hyperactivity [9]. Another cross-sectional study conducted in 106 American adolescents with an average age of 13 years old reported that adolescents at risk for psychological maladjustment were those who reported having poor sleep quality, such as sleep-wake problems and low sleep efficiency, measured by both actigraphy and subjective report [10]. Finally, another cross-sectional study based on a population of 10,000 Japanese adolescents in high school students reported that short sleep duration and subjective sleep problems were associated with poor mental health status [11]. However, it is still unclear which sleep variable is more widely associated with a larger number of mental health problems, and which specific mental health problems associate with a higher number of sleep variables. Therefore, further large population-based studies to address this question are still needed.

Concerning the differences between boys and girls, there are only few studies that have investigated the impact of gender on sleep patterns in adolescence. Previous studies have shown that the mental health of girls is more likely to be affected by problematic sleep habits, compared to boys. For example, Danielsson et al. suggested that sleep onset latency and wake after sleep onset measured by self-reported questionnaire among adolescents aged 16–18 years old were related to increased rate of depression in girls after puberty [12], but not in boys. In addition, Conklin suggested that chronic sleep deprivation measured by self-reported questionnaire among adolescents aged 13–18 years old increased the risk of major depression among young women [13]; and Rajab demonstrated an association between sleep measured by self-reported questionnaire and mental health including depressive symptoms and severe stress, in which severe stress was negatively associated with sleep in girls but not in boys among adolescents in grade 7–12 [14]. Finally, Lewien suggested that sleep-related difficulties are more frequent among girls than in boys in adolescents aged 10–17 years old, measured by self-reported questionnaires [15].

Although the association between sleep and some mental health problems in adolescence, such as depression and anxiety are relatively well established, there are still scarce studies investigating which sleep variables associate more widely with a higher number of mental health problems, and also which are the most affected mental health areas. Understanding the impact that specific sleep problems exert on several mental health problems in adolescence would contribute to the design of more effective interventions to improve adolescents’ mental health [1]. To fill the gap of the current literature, the objective of this study was to investigate the link between specific subjective self-reported sleep patterns (i.e., sleep duration, bedtime, frequency of night awakenings and sleep-onset latency) and several mental health problems, in particular developing affective symptoms, and emotional and behavioural disturbances in adolescence. More specifically, the main purpose was to examine how subjective sleep problems may affect these different developmental aspects in adolescence at the age of 13–14 years old, from a large population-based study, investigating whether sleep is more highly related to specific mental health areas, and understanding which sleep variables associate with a higher number of mental health problems, and which are the most affected mental health areas. We also analysed the associations between subjective sleep patterns and mental health separately in boys and girls, to understand the specific role that gender might exerts in the associations between sleep and mental health in adolescence. Based on previous studies, we hypothesized that self-reported shorter sleep, higher frequency of night awakening, later bedtime and longer sleep-onset latency would be related to several mental health difficulties such as anxiety and depressive symptoms [2] in adolescents at the age of 13–14 years old. Further, self-reported shorter sleep and higher frequency of night awakening would have a greater negative impact on mental health problems, compared to later bedtime and/or longer sleep onset latency; in addition, depression would be the mental health area greatly affected by sleep problems in adolescence.

## 2. Materials and Methods

### 2.1. Participants

This study is based on the Millennium Cohort Study (MCS) [16], which consists of a longitudinal birth cohort comprising 18,818 children born in the UK in 2000–2001, and who were followed up from birth until the age of 17 years old. The MCS is a British multi-disciplinary cohort that follows up children through their early childhood years into adolescence and adulthood [16]. Participating families were selected from a random sample with a stratified sampling design, to ensure representation throughout England, Scotland, Wales, and Northern Ireland. The MCS collects information from several areas such as parenting, childcare, general health, mental health, schooling, and education, among others, directly from the children (cohort members) and/or their parents or caregivers.

For this specific cross-sectional study, we focused only on the single time point of 13–14 years old, corresponding to the adolescence period. Cohort members at the age of 13–14 years old and their parents or caregivers were interviewed at home during the visit. Cohort members completed several self-reported questionnaires, which mainly explored wellbeing, sleep, mental health [17], mood, and feelings [18]. Parents or caregivers answered the socioeconomic questions regarding the information of both the family and the cohort members. At the time point of 13–14 years old, the MCS cohort contained 11,884 members that completed all the questions. Three hundred and thirty-one initial cases were excluded because they did not participate in the survey, or the parents did not complete the written consent. Therefore, the final sample size of this study comprises 11,553 participants at the age of 13–14 years old. The study was approved by the NHS Research Ethnics Committee, and written informed consent was obtained from all the parents and the young members themselves [19].

### 2.2. Measures

#### 2.2.1. Measures of Self-Reported Sleep Variables

The questions regarding sleep functioning in adolescence were developed by the MCS team [19], which are similar to the items used in other large longitudinal population-based studies [20]. Further, these sleep variables have been also used in recent research [21,22].

The sleep variables obtained from self-reported questionnaires [16] were: bedtime at night, wake up time in the morning, time to fall asleep during the last 4 weeks, and the frequency of night awakening during the last 4 weeks.

Concerning bedtime and wake up time, two time points for these sleep variables were available: weekdays (i.e., school days) and weekends (i.e., non-school days). For bedtime and wake up time, participants were asked: “What time do you usually go to sleep on a school night?”, “What time do you usually go to sleep on the nights when you do not have school the next day?”, “What time do you usually wake up in the morning on a school day?”, and “What time do you wake up in the morning on the days when you do not have school?”. For bedtime, the alternatives were: 1 = “before 9 p.m.”, 2 = “9–9:59 p.m.”, 3 = “10–10:59 p.m.”, 4 = “11-midnight”, and 5 = “after midnight”. For wake up time, the alternatives were: 1 = “before 8 a.m.”, 2 = “8–8:59 p.m.”, 3 = “9–9:59 p.m.”, 4 = “10–10:59 a.m.”, 5 = “11–11:59 a.m.”, 6 = “after midday”.

Furthermore, and for the purpose of this study, we created a variable of nighttime sleep duration, in hours, which was created as follows: bedtime minus wake up time; and this was defined for both weekdays and weekends.

Concerning sleep onset latency, participants were asked: “During the last four weeks, how long did it usually take for you to fall asleep?” The alternatives were: 1 = “0–15 min”, 2 = “16–30 min”, 3 = “31–45 min”, 4 = “46–60 min”, 5 = “more than 60 min”.

Finally, and for the frequency of night awakening, participants were asked: “During the last four weeks, how often did you awake during your sleep time and have trouble falling back to sleep again?” The adolescents rated their night awakenings on a scale ranging from 1 to 6, 1 = “All of the time”, 2 = “Most of the time”, 3 = “A good bit of the time”, 4 = “Some of the time”, 5 = “A little of the time”, 6 = “None of the time”.

#### 2.2.2. Measures of Mood, and Emotional and Behavioural Symptoms

Cohort members at the age of 13–14 years old completed the Mood and Feelings–short version (SMFQ), which includes 13 items on affective symptoms in the last 2 weeks [18]. The SMFQ shows relatively high internal reliability (Cronbach’s alpha = 0.85), 60% sensitivity and 85% specificity with a cut off score of 8 or more [18]. Sum scores for these items were calculated and this was the measure used for this study. Higher sum scores indicate higher prevalence of affective symptoms (i.e., depression).

Concerning emotional and behavioural functioning, parents or caregivers completed the Strengths and Difficulties Questionnaire (SDQ) [23], which is a screening questionnaire used to assess cohort member’s emotions and behaviour, including 25 items which are divided into 5 scales: (1) emotional symptoms, (2) hyperactivity/inattention, (3) conduct problems, (4) peer relationship problems and (5) prosocial behaviour. For this study, we focused on the total scores of emotional symptoms, conduct problems, and hyperactivity/inattention. Further, the SDQ total difficulties score was also included. SDQ shows Cronbach’s alpha values for the total difficulties score of 0.77 for the parents and 0.81 for the teachers, which is considered generally a high value [24].

#### 2.2.3. Confounders

Following previous research [4,6], these self-reported confounders were selected: gender (i.e., boy/girls), how close is the cohort member with mother/father, general health level, and alcohol consumption in the last four weeks. We selected each of these confounders based on the impact that each of these variables exert on mental health in adolescence. For instance, there is evidence suggesting that girls have worse mental health [25], optimal bonding with parents is associated with general well-being in adolescence [26], physical health associates with mental health in adolescence [27], and alcohol use is consistently associated with mental health problems in adolescents [28]. In relation to gender, considering that this is a variable that potentially interacts with the relationship between sleep variables and mental health outcomes, we initially included gender as a covariate in the initial models, and we then examined it directly in a subsequent set of models.

### 2.3. Statistical Analysis

Statistical analysis was performed with SPSS Statistics V26.0 (IBM, Armonk, NY, USA). Methods of statistical analysis included descriptive analyses, correlations, regression analysis, and path analysis. Descriptive statistics were conducted to obtain the means, standard deviations, frequencies, and percentages of the main variables. Pearson correlations were calculated to study the initial correlations between each of the main variables. To assess the specific associations between each of the sleep variables and mental health areas, such as affective symptoms, emotional symptoms, hyperactivity/inattention, and conduct problems in adolescents, linear regression analyses were further applied. Firstly, we conducted unadjusted models without covariates; then we conducted the adjusted model with covariates, including gender, the close level between cohort member and mother/father, general health level and alcohol consumption in the last four weeks. In addition, to investigate the differences between boys and girls, we divided the sample by gender, and conducted the linear regression analysis again. Finally, and to examine which sleep variables associated with a higher number of mental health problems, and to determine which were the strongest associations that remained after including all the sleep problems and main outcomes together, path analysis was conducted using AMOS v-27 package from SPSS.

## 3. Results

### 3.1. Descriptive Variables

Socio-demographic, sleep, and mental health variables of the cohort members are described in Table 1. Cohort members were aged between 13–14 years old (Mean: 13.77, SD: 0.45). The gender proportion of cohort members was relatively balanced: 50.1% of the sample were boys and 49.94% were girls. Further, 81.4% of the sample reported being very or extremely close with their mother/father. Only 4611 cohort members reported their status of drinking alcohol and half of them reported never drinking alcohol. Finally, 87.3% of the sample reported having good health. Other details of sleep variables, and mental health measures are further described in Table 1.

### 3.2. Correlation Results

The correlation analyses were conducted between all the sleep variables, including bedtime at school days, nighttime sleep hours at school days, bedtime at weekends, nighttime sleep hours at weekends, night awakening, and sleep-onset latency, and all the mental health outcomes (see Table 2). Interestingly, there was a moderate positive relationship (r > 0.3) between developing affective symptoms measured with SMFQ and frequency of night awakening (r = 0.300; *p* < 0.001) and sleep-onset latency (r = 0.378; *p* < 0.001). The other significant correlations were within the low ranges (r < 0.3), indicating a weak association (see Table 2).

### 3.3. Linear Regression Results

The results obtained from the linear regression models can be found in Table 3. Model A was unadjusted model without covariates; and model B was adjusted model with all the covariates, including gender, how close cohort member is with mother/father, general health level and alcohol consumption in the last four weeks.

In both models, higher frequency of night awakening was prospectively related to all mental health outcomes, including greater SMFQ affective symptoms (β = 0.302 in model A, β = 0.242 in model B), SDQ emotion symptoms (β = 0.166 in model A, β = 0.140 in model B), SDQ hyperactivity or inattention problems (β = 0.125 in model A, β = 0.121 in model B), SDQ conduct problems (β = 0.134 in model A, β = 0.097 in model B), and SDQ total difficulties (β = 0.176 in model A, β = 0.155 in model B, *p* < 0.001 for all measures). Further, longer sleep onset latency was related to higher scores in SMFQ affective symptoms (β = 0.164 in model A, β = 0.140 in model B, *p* < 0.001 in both models), SDQ emotional symptoms (β = 0.061 in model A, β = 0.065 in model B, *p* < 0.001 in both models), SDQ conduct problems (β = 0.029, *p* = 0.005 in model A, β = 0.038, *p* = 0.016 in model B), and SDQ total difficulties (β = 0.059 in model A, β = 0.061 in model B, *p* < 0.001 in both models).

Furthermore, later bedtime at school days was associated with greater SMFQ affective symptoms (β = 0.052, *p* < 0.001 in model A, β = 0.042, *p* = 0.046 in model B) and SDQ hyperactivity/inattention problems (β = 0.067, *p* < 0.001 in model A, β = 0.056, *p* = 0.025 in model B), and SDQ conduct problems (β = 0.045, *p* = 0.007 in model A, β = 0.055, *p* = 0.030 in model B). In relation to sleep duration, shorter nighttime sleep duration at school days was associated with greater SMFQ affective symptoms (β = −0.129 in model A, β = −0.068 in model B, *p* < 0.001 in both models), and higher scores in SDQ hyperactivity or inattention problems (β = 0.068, *p* < 0.001 in model A, β = 0.059, *p* = 0.009 in model B). Finally, shorter nighttime sleep duration at weekends was associated with greater SDQ hyperactivity/inattention problems (β = 0.027, *p* = 0.005 in model A, β = 0.038, *p* = 0.011 in model B), as well as greater SDQ conduct problems (β = 0.039, *p* = 0.001 in model A, β = 0.042, *p* = 0.005 in model B), and SDQ total difficulties (β = 0.030, *p* = 0.002 in model A, β = 0.041, *p* = 0.006 in model B).

#### Linear Regression Results: Gender Difference

In both boys and girls, night awakening was related to all the mental health outcomes (*p* < 0.001 in model A and model B).

For boys, the most noticeable finding was that later bedtime during school days associated with higher scores of SDQ emotional symptoms (β = 0.052, *p* = 0.023 in model A, β = 0.095, *p* = 0.008 in model B), SDQ hyperactivity/inattention (β = 0.061, *p* = 0.007 in model A, β = 0.116, *p* = 0.001 in model B), SDQ conduct problems (β = 0.055, *p* = 0.016 in model A, β = 0.098, *p* = 0.006 in model B), and SDQ total difficulties (β = 0.055, *p* = 0.015 in model A, β = 0.107, *p* = 0.002 in model B). However, in girls, later bedtime during school days was only related to greater SMFQ affective symptoms (β = 0.097, *p* < 0.001 in model A, β = 0.066, *p* = 0.031 in model B). This suggests that boys, who tend to go to bed later during school days, might be at higher risk for developing a broader range of mental health problems, including emotional and behavioural difficulties, compared to girls.

For girls, four sleep variables such as later bedtime at school days (β = 0.097, *p* < 0.001 in model A, β = 0.066, *p* = 0.033 in model B), shorter sleep duration at school days (β = −0.106, *p* < 0.001 in model A, β = −0.066, *p* = 0.031 in model B), higher night awakening frequency (β = 0.301, *p* < 0.001 in model A, β = 0.250, *p* < 0.001 in model B) and longer sleep onset latency (β = 0.164, *p* < 0.001 in model A, β = 0.147, *p* < 0.001 in model B) were all associated with greater SMFQ affective symptoms. This suggests that girls with problematic sleep habits are more likely to have higher risk of developing depression. The linear regression tables in relation to boys and girls are reported in Appendix A.

### 3.4. Path Analysis Results

A path model analysis was conducted in SPSS AMOSv-27 to confirm the associations obtained in the regression analyses among the whole sample, as well as to control for the potential effect of each mental health outcome with each other in adolescence. In adolescence, there is specially a high comorbidity of mental health outcomes, and by implementing path analyses we are controlling for the potential overlay of other mental health outcomes. Therefore, we aimed to investigate the effect of each sleep variable on specific mental health outcomes by controlling for the potential overlapping effect of comorbid mental health outcomes.

Based on the adjusted linear regression analysis results within the whole sample, we selected bedtime during school times, nighttime sleep hours during school times, nighttime sleep hours during the weekends, night awakening frequency, and sleep onset latency. As covariates, we included gender, wellbeing, how close the cohort member is with mother/father, and alcohol consumption.

**Figure 1:**
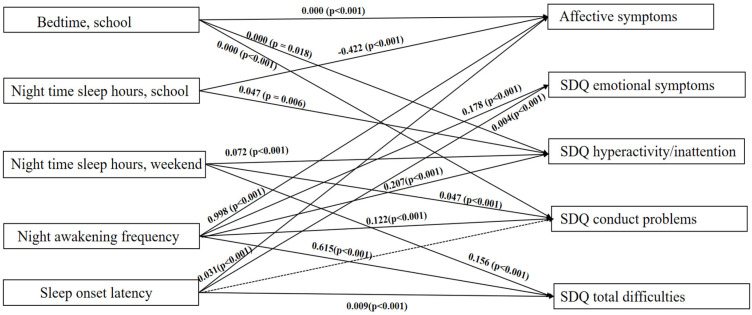
Path Diagram of Direct Associations in the Final Model. For clarity, pathways of the covariates with sleep variables and dependent variables, as well as association between the outcomes are not shown. Path analysis model fit indices indicated good model fit: chi-square = 10.612, *p* = 0.101; RMSEA = 0.008; CFI = 1.00. Significant pathways are represented by solid arrows; and nonsignificant modelled pathways represented by dotted lines. Bedtime at school days, nighttime sleep hours at school days, nighttime sleep hours at weekends, night awakening frequency, and sleep onset latency represent the independent variables (exposures), and affective symptoms, SDQ emotional symptoms, SDQ hyperactivity/inattention, SDQ conduct problems and SDQ total difficulties represent the dependent variables (outcomes). The covariates included also in this path analysis were gender, how close CM with mother/father, and alcohol consumption. The significant direct associations between the covariates and the independent and dependent variables were: gender and wellbeing with all dependent variables (all *p* < 0.001); how close adolescent with mother/father with affective symptoms (−0.748, *p* < 0.001), SDQ emotional symptoms (0.149, *p* = 0.005), SDQ hyperactivity/inattention (−0.118, *p* < 0.001), SDQ conduct problems (−0.203, *p* < 0.001), and SDQ total difficulties (−0.507, *p* = 0.003); and alcohol consumption with affective symptoms (0.273, *p* = 0.007), and SDQ emotional symptoms (−0.112, *p* = 0.002). SDQ = strength and difficulties questionnaire.

We modelled direct associations between sleep variables and five mental health outcome variables: Affective symptoms, SDQ emotional symptoms, SDQ hyperactivity/inattention, SDQ conduct problems, and SDQ total difficulties (see Figure 1). Path analysis model fit indices indicated good model fit: Chi-square = 10.612, *p* = 0.101; Root Mean Square Error of Approximation (RMSEA) = 0.008; Comparative Fit Index (CFI) = 1.00. Consistent with the adjusted linear regression analysis, higher night awakening frequency was significantly associated with all the mental health outcomes. Later bedtime during school time was significantly associated with three outcomes, including affective symptoms, SDQ hyperactivity/inattention, and SDQ conduct problems. Shorter nighttime sleep hours during school times were associated with affective symptoms, and SDQ hyperactivity. Shorter nighttime sleep hours during weekends were associated with SDQ hyperactivity/inattention, conduct problems, and SDQ total difficulties.

Longer sleep onset latency was significantly associated with four outcomes (i.e., all mental health outcomes except for SDQ hyperactivity/inattention), which were also consistent with the regression analyses. There was some difference between the linear regression and the path analysis results. In the linear regression analysis, longer sleep onset latency was associated with four outcomes, including affective symptoms, SDQ emotional problems, SDQ conduct problems, and SDQ total difficulties. However, in the path analysis, longer sleep onset latency was associated with three outcomes, including affective symptoms, SDQ emotional symptoms, and SDQ total difficulties, but not with SDQ conduct problems.

## 4. Discussion

### 4.1. Principal Findings

Our main findings showed that higher night awakening frequency associates with a higher number of mental health problems in adolescents at the age of 13–14 years old, followed by longer sleep onset latency and shorter night-time sleep hours, which were also associated with several mental health problems in adolescence. Among the mental health areas investigated in this study, hyperactivity/inattention was the dimension that associated with a higher number of sleep problems.

First, we found that higher frequency of night awakening was associated with all the mental health outcomes included in this study (i.e., affective symptoms, emotional problems, conduct problems, hyperactivity/inattention problems and mood and behavioural difficulties). Second, longer sleep onset latency, later bedtime during school days and short nighttime sleep duration during school days were all associated with affective symptoms. Further, longer sleep onset latency was also associated with emotional symptoms and overall mental health difficulties. Third, shorter nighttime sleep hours during weekends were associated with hyperactivity/inattention problems and overall mental health difficulties.

Importantly, we found different patterns of associations between school days and weekends in some sleep variables in the whole sample. From the descriptive analysis, we observed that adolescents aged 13–14 years old usually go to sleep later and sleep for longer hours during the weekends, probably due to the school scheduling. In relation to bedtime, although we did not find any significant association between later bedtime during the weekends and any of the mental health outcomes, we found that later bedtime during school days was associated with affective symptoms and hyperactivity/inattention. Concerning nighttime sleep duration, although shorter nighttime sleep duration during school days and weekends were both associated with hyperactivity/inattention, shorter nighttime sleep duration during school days was additionally associated with affective symptoms, while shorter nighttime sleep duration during weekends was additionally associated with conduct problems and overall mental health difficulties.

Our main findings are consistent with previous research, where sleep deprivation exerted modulatory influence on the onset of psychiatric conditions, including depression and emotional regulation in adolescents [29]. Further, in a previous Japanese longitudinal study using a population of 516 junior high school teenagers, the authors indicated that a new onset of sleep disturbance or persistent sleep disturbance during two consecutive years could be a risk factor for the development of poorer mental health [4]. The findings of our current study were consistent with this suggestion, as higher frequency night awakening, longer sleep onset latency, later bedtime during school days, and shorter sleep hours were all associated with affective symptoms and mental health difficulties in adolescence. Our findings were also consistent with other previous research, where shortened sleep duration and impaired sleep quality associated with mental health problems, such as anxiety and externalizing symptoms in early adolescents [1]. In addition, Matamura et al. reported a significant association between late bedtime and mental health disturbance, such as depression, anxiety, and suicidal or self-injury risk in monozygotic twin adolescents [8].

However, most of the previous studies have focused on nighttime sleep duration, while there is still less research conducted on the impact that night awakening and/or sleep onset latency might exert on mental health problems in adolescence. Therefore, our current study contributes with the findings that night awakening and sleep onset latency are associated with the most relevant mental health problems in adolescence, including affective symptoms, emotional difficulties, mood, and behavioural difficulties.

### 4.2. Gender Differences

The secondary analyses of this study investigated the potential gender differences in the associations between sleep and mental health among adolescents. First, we found gender-specific differences in our study, with boys’ mental health and behavioural problems being affected by the vast majority of the sleep problems included in this study, especially later bedtime, while in girls, late bedtime, nighttime sleep duration during school days, night awakening frequency and long sleep onset latency all associated with affective symptoms. Therefore, this suggests that poor sleep seems to manifest more externally in males, while more internally in females.

Concerning night awakening frequency, we found that higher number of night awakening was related to all the mental health outcomes in both boys and girls, which follows the same pattern that we observed in the whole sample. However, we also found gender differences in the associations between sleep and mental health outcomes in adolescence, as reported above.

Contrary to previous studies, which suggest that girls have poorer mental health and lower sleep scores compared to boys [30], we found that sleep disturbances had a greater negative impact on mental health among boys than among girls. This is a novelty finding, since boys with sleep disturbance (i.e., late bedtime, short nighttime sleep hours during school days and weekends, night awakening frequency, sleep onset latency), were more likely to suffer from a wider range of mental health difficulties, including hyperactivity and conduct problems, compared to girls.

Our findings that girls with sleep difficulty (i.e., late bedtime during school days, shorter sleep hours at school days, night wakening and long sleep onset latency) present higher risk of developing affective disorders compared to boys is consistent with the emerging literature on the topic. For instance, Danielsson et al. [12] suggested that longer sleep onset latency and later wake after sleep onset were related to an increased rate of depression in girls after puberty. Further, Conklin found that chronic sleep deprivation increases the risk of major depression in girls after puberty [13].

### 4.3. Study Strengths

The most noticeable strength of this study was the large sample size and the coverage of several sleep measures. Also, it is a large population-based study and it is representative of the UK population., Therefore, the information and main findings can be used as reference values for clinical practitioners in the UK. In addition, we focused on a narrow age band (i.e., 13–14 years old) in adolescence.

### 4.4. Study Limitations

First, although we controlled for relevant confounding factors such as gender, how close is the cohort member with mother/father, general health level, and alcohol consumption in the last four weeks, there are still other environmental, developmental and parental factors that may have contributed to the associations between sleep and mental health, such as social media use [31], video devices use time, night activities [32], and body mass index [33]. In addition, other relevant socio-demographic factors, including ethnicity, level of education or economic status could have also impacted our results. Second, self-reporting and parent-reported adolescent’s information tends to be subjective. Compared to more objective sleep measures like actigraphy, participants using self-reporting measures may have overestimated or underestimated sleep. Therefore, future studies should consider the inclusion of more objective sleep measures, such as actigraphy. Third, the current measures of sleep, such as bedtime and wake time were collected by categorical variables, in which individual stated the hour in which they went to bed rather than the specific time, which only provided a relatively broad index of sleep duration. Fourth, the self-reported sleep variables used in this study have not been validated, thus psychometric properties of these items are not available. Fifth, a further limitation is that our findings did not fully cover the situation for the vulnerable families; for example, we did not include the family exposure to extreme negative and chaotic situation, such as those family suffering from crime, disaster, etc. Finally, overall, most of the correlations were weak, thus this should be cautiously taken into account when interpreting these results.

## 5. Conclusions

Our study found that higher night awakening frequency associates with higher number of mental health problems in adolescents at the age of 13–14 years old. Also, we found that hyperactivity/inattention associates with higher number of sleep problems in adolescents at the age of 13–14 years old.

This study contributes to the recent research in the field of sleep problems in adolescents, by showing not only that sleep duration, but also frequent night awakening and long sleep onset latency are all related to several mental health problems in adolescence. Further, the association between sleep and mental health may vary between boys and girls. For instance, poor sleep seems to manifest more externally in males, while more internally in females.

Clinical practitioners and parents will benefit from these findings, as these suggest that specific sleep problems should be considered when addressing adverse mental health problems in adolescents. This would allow clinicians to develop more targeted prevention and intervention strategies in adolescents’ mental health. In addition, gender differences should be also considered when addressing mental health in adolescents.

## Figures and Tables

**Table 1:** Descriptive statistics of socio-demographic, sleep, and mental health variables in adolescence.

	Sociodemographic Variables		
	N	Frequency	%
Sex (Male/Female)	11,884	5953/5931	50.1/49.94
	N	Mean	SD
Age, in years	11,884	13.77	0.45
	**Sleep Variables**		
	N	Mean	SD
Bedtime school time, hh:mm	11,479	22:26	0:51
Wakeup school time, hh:mm	11,491	7:03	0:39
Nighttime sleep hours school	11,496	8.63	0.97
Nighttime sleep hours at weekends	11,485	10.69	1.22
Bedtime weekends/holidays	11,482	23:17	0:44
Wakeup time weekends/holidays	11,478	9:59	1:18
Sleep onset latency (minutes)	11,420	27.12	19.65
Night awakening frequency (during last 4 weeks)	11,457	1.39	1.37
	**Variables of Mental Health**		
	N	Mean (Min/Max)	SD
Total score of SMFQ	11,200	18.52 (13/39)	5.85
SDQ Emotional Symptoms	11,486	2.05 (0/10)	2.14
SDQ Conduct Problems	11,488	1.42 (0/10)	1.63
SDQ Hyperactivity/Inattention	11,481	2.99 (0/10)	2.40
SDQ Peer Problems	11,491	1.74 (0/10)	1.82
SDQ Prosocial	11,489	8.31 (0/10)	1.85
SDQ Total Difficulties	11,471	8.19 (0/38)	5.99

CM = cohort members; SMFQ = Short Mood and Feelings Questionnaire; SDQ = The Strengths and Difficulties Questionnaire.

**Table 2:** Correlations between sleep variables and mental health in adolescents.

	Bedtime School		Nighttime Sleep Hours, School		Bedtime Weekends		Nighttime Sleep Hours, Weekends		Night Awakening		Sleep-Onset Latency, Mins	
	r	*p*	r	*p*	r	*p*	r	*p*	r	*p*	r	*p*
Total Feelings	**0.230 ^a^**	**<0.001**	**−0.248 ^a^**	**<0.001**	**0.168 ^a^**	**<0.001**	**−0.031 ^a^**	**0.001**	**0.300 ^b^**	**<0.001**	**0.378 ^b^**	**<0.001**
SDQ Emotional Symptoms	**0.043 ^a^**	**<0.001**	**−0.44 ^a^**	**<0.001**	**0.019 ^a^**	**0.047**	0.011	0.245	**0.188 ^a^**	**<0.001**	**0.118 ^a^**	**<0.001**
SDQ Hyperactivity/Inattention	**0.053 ^a^**	**<0.001**	−0.012	0.209	**0.056 ^a^**	**<0.001**	**0.023 ^a^**	**0.016**	**0.131 ^a^**	**<0.001**	**0.068 ^a^**	**<0.001**
SDQ Conduct Problems	**0.088 ^a^**	**<0.001**	**−0.062 ^a^**	**<0.001**	**0.085 ^a^**	**<0.001**	**0.026 ^a^**	**0.006**	**0.150 ^a^**	**<0.001**	**0.088 ^a^**	**<0.001**
SDQ Peer Problems	0.010	0.297	−0.010	0.308	−0.011	0.237	0.017 ^a^	0.076	**0.120 ^a^**	**<0.001**	**0.100 ^a^**	**<0.001**
SDQ Total Difficulties	**0.064 ^a^**	**<0.001**	**−0.040 ^a^**	**<0.001**	**0.049 ^a^**	**<0.001**	**0.025 ^a^**	**0.008**	**0.198 ^a^**	**<0.001**	**0.125 ^a^**	**<0.001**

CM = cohort members; Total feelings: Mood and Feelings–short version (SMFQ); SDQ: the Strengths and Difficulties Questionnaire; ^a^ Weak correlations: r < 0.3; ^b^ Moderate correlations: r ≥ 0.3. Bold: *p* < 0.05.

**Table 3:** Unadjusted and Adjusted Associations Between Sleep Variables and Mental Health variables in adolescents at 13–14 Years Old.

	Model A			Model B		
Sleep Variables	β	*p* Values	95.0% CI for B	β	*p* Values	95.0% CI for B
**SMFQ**						
**Bedtime school**	**0.052**	**<0.001**	**0.000 to 0.000**	**0.042**	**0.046**	**0.000 to 0.000**
**Nighttime sleep hours, school**	**−0.129**	**<0.001**	**−0.945 to −0.632**	−0.068	**<0.001**	**−0.710 to −0.213**
Bedtime weekends	**0.039**	**<0.001**	**0.000 to 0.000**	0.004	0.770	0.000 to 0.000
Nighttime sleep hours, weekends	−0.004	0.680	−0.100 to 0.065	−0.016	0.203	−0.218 to 0.041
**Night awakening**	**0.302**	**<0.001**	**1.229 to 1.380**	**0.242**	**<0.001**	**1.005 to 1.247**
**Sleep onset latency, mins**	**0.164**	**<0.001**	**0.044 to 0.054**	**0.140**	**<0.001**	**−0.037 to 0.053**
**SDQ Emotional symptoms**						
Bedtime school	0.021	0.191	0.000 to 0.000	0.010	0.525	0.000 to 0.000
Nighttime sleep hours, school	−0.009	0.535	−0.082 to 0.043	−0.009	0.685	−0.113 to 0.075
Bedtime weekends	−0.016	0.173	0.000 to 0.000	0.001	0.950	0.000 to 0.000
Nighttime sleep hours, weekends	0.012	0.217	−0.012 to 0.054	0.020	0.178	−0.016 to 0.084
**Night awakening**	**0.166**	**<0.001**	**0.227 to 0.287**	**0.14** **0**	**<0.001**	**0.164 to 0.256**
**Sleep onset latency, mins**	**0.061**	**<0.001**	**0.004 to 0.009**	**0** **.065**	**<0.001**	**0.004 to 0.010**
**SDQ Hyperactivity/inattention**						
**Bedtime school**	**0.067**	**<0.001**	**0.000 to 0.000**	**0.056**	**0.025**	**0.000 to 0.000**
**Nighttime sleep hours, school**	**0.068**	**<0.001**	**0.097 to 0.238**	**0.059**	**0.009**	**−0.037 to 0.257**
Bedtime weekends	**0.043**	**<0.001**	**0.000 to 0.000**	0.019	0.289	0.000 to 0.000
**Nighttime sleep hours, weekends**	**0.027**	**0.005**	**0.016 t0 0.090**	**0.038**	**0.011**	**0.018 to 0.133**
**Night awakening**	**0.125**	**<0.001**	**0.182 to 0.250**	**0.121**	**<0.001**	**0.153 to 0.259**
Sleep onset latency, mins	0.018	0.068	0.000 to 0.005	0.014	0.376	−0.002 to 0.005
**SDQ conduct problems**						
**Bedtime school**	**0.045**	**0.007**	**0.000 to 0.000**	**0.055**	**0.030**	**0.000 to 0.000**
Nighttime sleep hours, school	0.009	0.535	−0.032 to 0.062	0.022	0.321	−0.038 to 0.115
Bedtime weekends	**0.056**	**<0.001**	0.000 to 0.000	0.034	0.063	0.000 to 0.000
**Nighttime sleep hours, weekends**	**0.039**	**<0.001**	**0.026 to 0.076**	**0.042**	**0.005**	**0.018 to 0.098**
**Night awakening**	**0.134**	**<0.001**	**0.134 to 0.179**	**0.097**	**<0.001**	**0.079 to 0.153**
**Sleep onset latency, mins**	**0.029**	**0.005**	**0.001 to 0.004**	**0.038**	**0.016**	**0.001 to 0.006**
**SDQ Total difficulties**						
Bedtime school	**0.049**	**0.003**	**0.000 to 0.000**	0.042	0.091	0.000 to 0.000
Nighttime sleep hours, school	0.027	0.059	−0.007 to 0.338	0.022	0.327	−0.132 to 0.396
Bedtime weekends	0.017	0.146	0.000 to 0.000	0.016	0.380	0.000 to 0.000
**Nighttime sleep hours, weekends**	**0.030**	**0.002**	**0.056 to 0.238**	**0.041**	**0.006**	**0.056 to 0.335**
**Night awakening**	**0.176**	**<0.001**	**0.674 to 0.840**	**0.155**	**<0.001**	**0.519 to 0.777**
**Sleep onset latency, mins**	**0.059**	**<0.001**	**0.012 to 0.024**	**0.061**	**<0.001**	**0.008 to 0.026**

Model A = Unadjusted model; Model B = Adjusted for How close is CM with mother/father, During last 4 weeks how many times CM has had an alcoholic drink, General level of health. CI = Confidence Interval; CM = cohort member. Bold: *p* < 0.05.

## Data Availability

The data can be requested from the authors.

## Appendix A

**Table A1:** Unadjusted and Adjusted Associations Between Sleep Variables and Mental Health variables in boys at 13–14 Years Old.

	Model A			Model B		
Sleep Variables	β	*p* Values	95.0% CI for B	β	*p* Values	95.0% CI for B
**SMFQ**						
Bedtime school	0.052	0.016	0.000 to 0.000	0.018	0.589	0.000 to 0.000
**Nighttime sleep hours, school**	**−0.088**	**<0.001**	**−0.600 to −0.242**	**−0.074**	**0.011**	**−0.683 to −0.090**
Bedtime weekend	0.009	0.575	0.000 to 0.000	0.009	0.703	0.000 to 0.000
Nighttime sleep hours, weekend	−0.018	0.173	−0.158 to 0.028	−0.028	0.157	−0.269 to 0.043
**Night awakening**	**0.255**	**<0.001**	**0.816 to 0.998**	**0.241**	**<0.001**	**0.791 to 1.095**
**Sleep-onset latency, mins**	**0.158**	**<0.001**	**0.031 to 0.043**	**0.152**	**<0.001**	**0.028 to 0.048**
**SDQ Emotional symptoms**						
**Bedtime school**	**0.052**	**0.023**	**0.000 to 0.000**	**0.095**	**0.008**	**0.000 to 0.000**
Nighttime sleep hours, school	0.038	0.060	−0.003 to 0.161	0.047	0.136	−0.028 to 0.209
Bedtime weekend	−0.013	0.430	0.000 to 0.000	−0.002	0.939	0.000 to 0.000
Nighttime sleep hours, weekend	0.009	0.511	−0.028 to 0.057	0.021	0.330	−0.031 to 0.093
**Night awakening**	**0.133**	**<0.001**	**0.163 to 0.246**	**0.112**	**<0.001**	**0.099 to 0.221**
**Sleep-onset latency, mins**	**0.054**	**<0.001**	**0.003 to 0.008**	**0.072**	**0.001**	**0.003 to 0.011**
**SDQ Hyperactivity/inattention**						
**Bedtime school**	**0.061**	**0.007**	**0.000 to 0.000**	**0.116**	**0.001**	**0.000 to 0.000**
**Nighttime sleep hours, school**	**0.045**	**0.027**	**0.013 to 0.219**	**0.071**	**0.024**	**0.024 to 0.343**
Bedtime weekend	0.012	0.488	0.000 to 0.000	−0.009	0.732	0.000 to 0.000
Nighttime sleep hours, weekend	0.034	0.014	0.013 t0 0.119	0.041	0.057	−0.002 to 0.164
**Night awakening**	**0.148**	**<0.001**	**0.233 to 0.338**	**0.137**	**<0.001**	**0.181 to 0.345**
Sleep-onset latency, mins	0.056	<0.001	0.004 to 0.011	0.042	0.055	0.000 to 0.010
**SDQ conduct problems**						
**Bedtime school**	**0.055**	**0.016**	**0.000 to 0.000**	**0.098**	**0.006**	**0.000 to 0.000**
Nighttime sleep hours, school	0.002	0.913	−0.065 to 0.072	0.023	0.748	−0.072 to 0.149
Bedtime weekend	0.024	0.156	0.000 to 0.000	−0.007	0.780	0.000 to 0.000
**Nighttime sleep hours, weekend**	**0.047**	**0.001**	**0.026 to 0.097**	**0.057**	**0.007**	**0.021 to 0.136**
**Night awakening**	**0.132**	**<0.001**	**0.135 to 0.204**	**0.101**	**<0.001**	**0.077 to 0.191**
**Sleep-onset latency, mins**	**0.057**	**<0.001**	**0.003 to 0.007**	**0.061**	**0.005**	**0.002 to 0.009**
**SDQ Total difficulties**						
**Bedtime school**	**0.055**	**0.015**	**0.000 to 0.000**	**0.107**	**0.002**	**0.000 to 0.000**
Nighttime sleep hours, school	0.030	0.138	−0.060 to 0.436	0.048	0.125	−0.082 to 0.6690
Bedtime weekend	−0.003	0.843	0.000 to 0.000	−0.013	0.620	0.000 to 0.000
**Nighttime sleep hours, weekend**	**0.036**	**0.009**	**0.043 to 0.299**	**0.051**	**0.017**	**0.043 to 0.434**
**Night awakening**	**0.164**	**<0.001**	**0.640 to 0.892**	**0.146**	**<0.001**	**0.474 to 0.859**
**Sleep-onset latency, mins**	**0.084**	**<0.001**	**0.018 to 0.035**	**0.085**	**<0.001**	**0.012 to 0.037**

Model A = Unadjusted model; Model B = Adjusted for How close is CM with mother/father, During last 4 weeks how many times CM has had an alcoholic drink, General level of health. CI = Confidence Interval; CM = cohort member. Bold: *p* < 0.05.

**Table A2:** Unadjusted and Adjusted Associations Between Sleep Variables and Mental Health variables in girls at 13–14 Years Old.

	**MODEL A**			**MODEL B**		
**Sleep Variables**	**β**	***p*** **Values**	**95.0% CI for B**	**β**	***p*** **Values**	**95.0% CI for B**
**SMFQ**						
**Bedtime school**	**0.097**	**<0.001**	**0.000 to 0.000**	**0.066**	**0.031**	**0.000 to 0.000**
**Nighttime sleep hours, school**	**−0.106**	**<0.001**	**−0.969 to −0.477**	**−0.067**	**0.015**	**−0.894 to −0.096**
Bedtime weekend	0.064	<0.001	0.000 to 0.000	−0.001	0.971	0.000 to 0.000
Nighttime sleep hours, weekend	−0.018	0.143	−0.232 to 0.033	−0.004	0.805	−0.243 to 0.189
**Night awakening**	**0.301**	**<0.001**	**1.294 to 1.522**	**0.250**	**<0.001**	**1.027 to 1.400**
**Sleep-onset latency, mins**	**0.164**	**<0.001**	**0.046 to 0.063**	**0.147**	**<0.001**	**0.038 to 0.064**
**SDQ Emotional symptoms**						
Bedtime school	0.019	0.419	0.000 to 0.000	−0.045	0.208	0.000 to 0.000
Nighttime sleep hours, school	−0.016	0.459	−0.129 to 0.058	−0.049	0.122	−0.262 to 0.031
Bedtime weekend	−0.019	0.261	0.000 to 0.000	−0.006	0.822	0.000 to 0.000
Nighttime sleep hours, weekend	0.003	0.830	−0.056 to 0.045	0.015	0.475	−0.050 to 0.108
**Night awakening**	**0.165**	**<0.001**	**0.214 to 0.301**	**0.155**	**<0.001**	**0.171 to 0.307**
**Sleep-onset latency, mins**	**0.059**	**<0.001**	**0.003 to 0.010**	**0.061**	**0.007**	**0.002 to 0.012**
**SDQ Hyperactivity/inattention**						
Bedtime school	0.020	0.403	0.000 to 0.000	−0.011	0.8768	0.000 to 0.000
Nighttime sleep hours, school	0.021	0.308	−0.044 to 0.140	0.047	0.147	−0.039 to 0.261
Bedtime weekend	0.082	<0.001	0.000 to 0.000	0.053	0.047	0.000 to 0.000
Nighttime sleep hours, weekend	**0.055**	**<0.001**	**0.053 t0 0.152**	0.032	0.136	−0.019 to 0.142
**Night awakening**	**0.159**	**<0.001**	**0.201 to 0.286**	**0.102**	**<0.001**	**0.088 to 0.228**
Sleep-onset latency, mins	−0.014	0.321	−0.005 to 0.002	−0.015	0.516	−0.007 to 0.003
**SDQ conduct problems**						
Bedtime school	0.016	0.515	0.000 to 0.000	0.007	0.846	0.000 to 0.000
Nighttime sleep hours, school	−0.006	0.778	−0.074 to 0.056	0.025	0.430	−0.063 to 0.148
**Bedtime weekend**	**0.095**	**<0.001**	**0.000 to 0.000**	**0.078**	**0.002**	**0.000 to 0.000**
Nighttime sleep hours, weekend	0.039	0.004	0.016 to 0.086	0.021	0.315	−0.028 to 0.086
**Night awakening**	**0.151**	**<0.001**	**0.133 to 0.193**	**0.086**	**<0.001**	**0.047 to 0.146**
Sleep-onset latency, mins	−0.002	0.907	−0.002 to 0.002	0.013	0.570	−0.002 to 0.004
**SDQ Total difficulties**						
Bedtime school	0.023	0.335	0.000 to 0.000	−0.025	0.475	0.000 to 0.000
Nighttime sleep hours, school	−0.003	0.894	−0.257 to 0.224	−0.001	0.975	−0.377 to 0.365
Bedtime weekend	0.041	0.018	0.000 to 0.000	0.043	0.095	0.000 to 0.000
Nighttime sleep hours, weekend	0.036	0.008	0.046 to 0.305	0.025	0.222	−0.076 to 0.325
**Night awakening**	**0.205**	**<0.001**	**0.719 to 0.941**	**0.155**	**<0.001**	**0.441 to 0.786**
**Sleep-onset latency, mins**	**0.034**	**0.019**	**0.002 to 0.018**	0.036	0.104	−0.002 to 0.022

Model A = Unadjusted model; Model B = Adjusted for How close is CM with mother/father, During last 4 weeks how many times CM has had an alcoholic drink, General level of health. CI = Confidence Interval; CM = cohort member. Bold: *p* < 0.05.
